# Intraoperative Fluoroscopy Exposure: A Systematic, Multi-specialty Review of Occupational Radiation Exposure to Surgeons

**DOI:** 10.7759/cureus.96001

**Published:** 2025-11-03

**Authors:** Ellie Helton, Kerilyn Godbe, Cole Bird, Jacob Brubacher

**Affiliations:** 1 Plastic Surgery, University of Kansas School of Medicine, Kansas City, USA; 2 Plastic Surgery, University of Kansas Medical Center, Kansas City, USA; 3 Orthopedic Surgery, University of Kansas Medical Center, Kansas City, USA

**Keywords:** fluoroscopy, intraoperative exposure, radiation, risk prevention, surgery

## Abstract

Fluoroscopy is commonly used intraoperatively to obtain real-time procedural imaging. While this technique is integral for patient care, it increases the exposure of surgeons and staff to ionizing radiation. To date, no comprehensive systematic review has examined the radiation risks associated with fluoroscopy or the protective strategies implemented across surgical subspecialties. Following PRISMA (Preferred Reporting Items for Systematic reviews and Meta-Analyses) guidelines, we searched PubMed and EMBASE to identify studies reporting surgeons’ intraoperative occupational radiation exposure and related health outcomes due to fluoroscopy use.

Of the 1,235 articles initially screened, 71 met the inclusion criteria and were subsequently grouped into six common themes: review articles (13/71, 18.3%), radiation exposure (13/71, 18.3%), radiation reduction techniques (other than shielding) (15/71, 21.1%), radiation shielding (9/71, 12.7%), cancer prevalence/risk (12/71, 16.9%), and guideline awareness and adherence (9/71, 12.7%).

Given the hazards of ionizing radiation, surgeons must understand the levels of exposure, associated risks, and preventive strategies when using intraoperative fluoroscopy. This multispecialty review synthesizes the current literature on occupational fluoroscopic radiation exposure and provides surgeons with the guidance needed to perform these procedures safely.

## Introduction and background

Ionizing radiation, radiation that has enough energy to ionize molecules by removing electrons, is a hazardous yet essential tool in modern surgical practice [1-19]. While X-rays have been used in medicine since 1895, the first widely recognized safety guidelines for working with radiologic materials were published by the International Commission on Radiological Protection (ICRP) in 1928 [3]. While original recommendations were based on dose resulting in observed skin erythema [3], modern guidelines have been extrapolated from the Life Span Study of survivors of the atomic bombings in Hiroshima and Nagasaki, and the Chernobyl accident [1,4]. Recommendations follow what is known as the “ALARA Principle” or “As Low As Reasonably Achievable”, meaning using the least amount of radiation necessary to get an image for the best outcome [20,21].

Currently, the ICRP recommends a maximum occupational radiation exposure of 20 millisieverts (mSv) per year (with 1 sievert corresponding to an effective dose of 1 J/kg of matter) [4], whereas the U.S. Nuclear Regulatory Commission (NRC) continues to uphold the 1956 annual limit of 50 mSv [20,21]. This is calculated as a dose area product (DAP), or the dose recorded on a dosimeter at a site and the amount of the surgeon’s body exposed [7]. This includes “scatter radiation”, which is energy from the X-ray that is not absorbed by tissue, but rather deflected to continue with a lower dose of energy [1]. For reference, the annual average background radiation dose of a U.S. citizen is 6.2 mSv (620 mrem) [20,21]. Sieverts, the Standard International (SI) unit, are used to measure the health effect, or energy absorbed per unit mass of ionizing radiation, where one mSv is equal to one mGy and 100 mrem [1,20,21].

Fluoroscopy is a form of X-ray imaging that is commonly used intraoperatively to obtain real-time, moving procedural imaging. When the X-ray interacts with tissues, atoms and molecules in that tissue “ionize” and can damage and kill tissues [1]. This allows physicians to observe tissues and organs in real time as they move. Although fluoroscopy is essential for patient care, it also increases occupational exposure to ionizing radiation for surgeons and operating room staff [1-19]. Without the use of lead protection, surgeons who frequently perform fluoroscopy-guided procedures can easily exceed the recommended annual limit of 20 mSv, with some studies reporting exposures up to three times this threshold [13]. Surgeons and surrounding operating room staff are exposed to ionizing radiation when the X-ray machine is “shooting”, often for a few seconds at a time [1]. Despite the proven effectiveness of shielding, noncompliance rates range from 10% [22] to 16% [23], varying by study population and type of protective equipment. Importantly, radiation safety training has been shown to significantly improve shielding compliance [24]. Nevertheless, even with proper lead protection, highly active surgeons may still surpass the 20 mSv annual guideline [5].

Recently, many studies have addressed surgeon radiation exposure, associated risks, and preventive strategies. Despite growing interest in this topic, no research to date has comprehensively consolidated these findings across all surgical subspecialties. Given the importance of education and knowledge on physician adherence and resultant radiation risk reduction, we aimed to create a reference summarizing the research in this field that is relevant to all practicing surgeons utilizing operative fluoroscopy [25,23,26]. This review is designed to provide busy surgeons of any specialty with the knowledge needed to protect themselves while optimizing patient outcomes.

## Review

Methods

**Figure 1:**
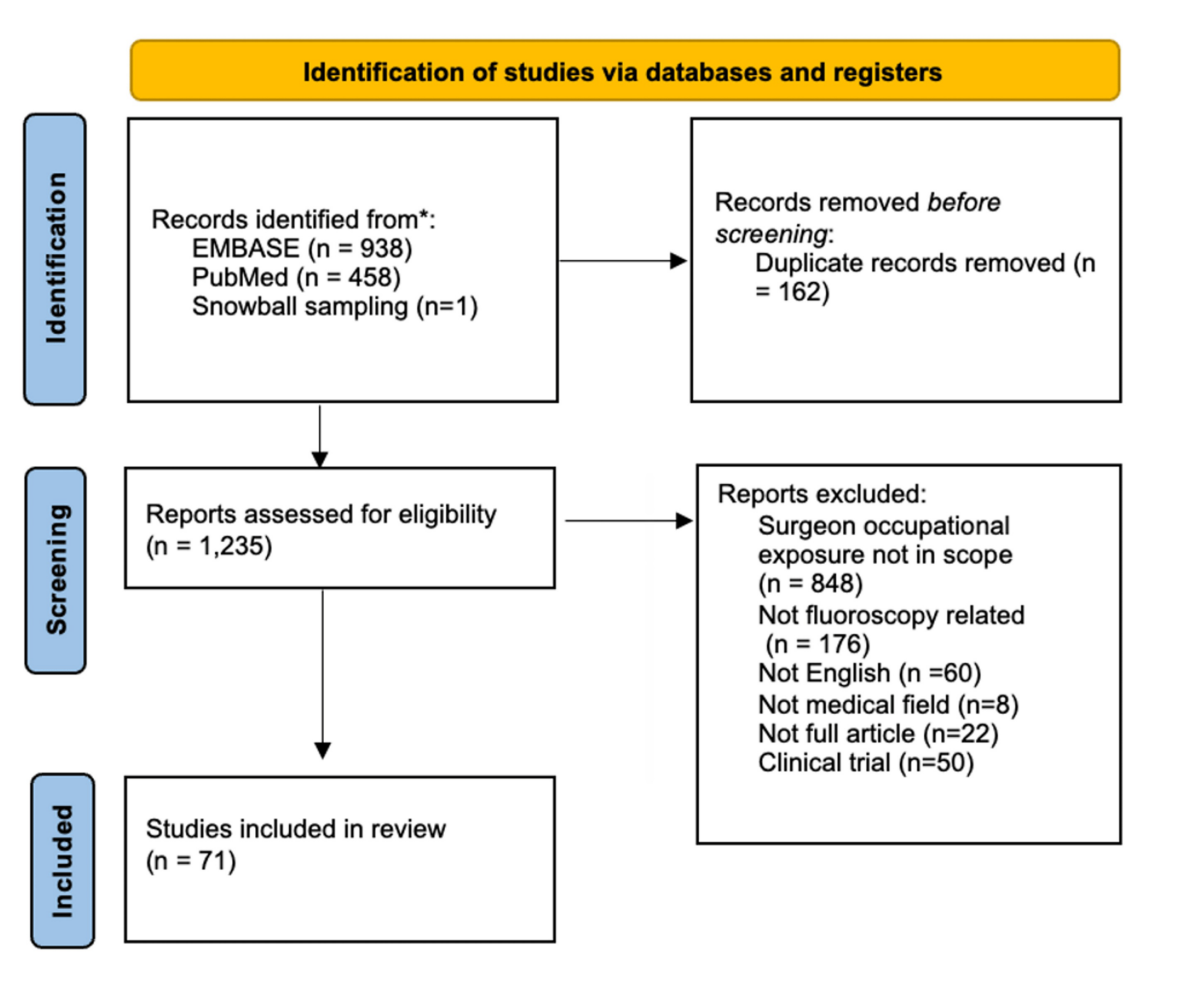
PRISMA flow chart depicting the selection of studies PRISMA: Preferred Reporting Items for Systematic reviews and Meta-Analyses

Per PRISMA (Preferred Reporting Items for Systematic reviews and Meta-Analyses) guidelines, a systematic literature review was performed using PubMed and EMBASE using the keywords “surgeon”, “exposure”, “radiation”, and “cancer” from January 1977 to October 2025 (Figure 1). This search yielded 458 and 938 articles, respectively. In addition to the initial database search, additional relevant articles were identified through snowball sampling, in which the reference lists of key studies were reviewed to capture important papers not retrieved by the original search terms. Inclusion criteria encompassed full articles, including reviews, written in English that addressed intraoperative fluoroscopy and occupational radiation exposure or related risks for operating surgeons, yielding a total of 71 articles. Studies were excluded if they did not focus on surgeon occupational exposure, were unrelated to fluoroscopy, were non-English or outside the medical field, or were not full articles.

**Figure 2:**
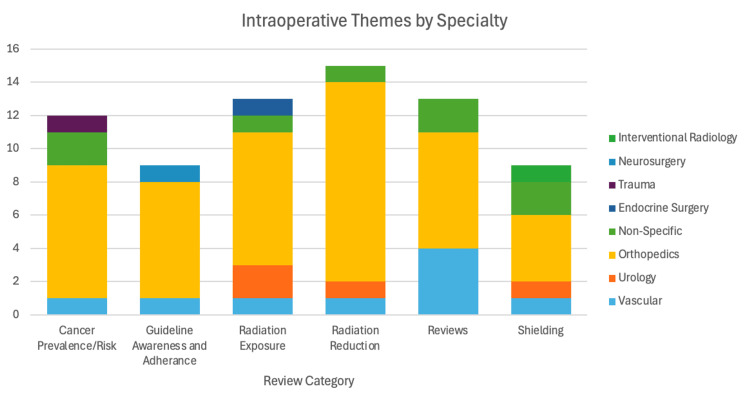
Article breakdown by specialty and theme

Articles were grouped into six categories: review articles, radiation exposure, radiation shielding, radiation reduction techniques (excluding shielding), cancer prevalence/risk, and surgeon awareness and education (Figure 2). Each category forms a subsection of this paper. The radiation exposure category examined exposure by body location, surgeon subspecialty, procedure type, and patient factors that influence higher radiation levels. Radiation shielding included all forms of intraoperative protective measures. Radiation reduction techniques focused on surgeon positioning, experience, and the use of operative equipment. The cancer prevalence and risk subsection summarized known associations between intraoperative radiation exposure and adverse outcomes, including cancer and congenital defects. Finally, the surgeon awareness and education category covered studies on radiation safety knowledge, training, and adherence.

Results

Review Articles

We identified 15 review articles on intraoperative radiation exposure, risk, and/or safety with fluoroscopy use (Table 1). Of these, 27% (3/15) focused on endovascular procedures, 36% (6/15) focused on orthopedic surgery cases, 18% (2/15) on spinal surgery, and 9% (1/15) on joint arthroscopy. Two review articles encompassed all surgical specialties but only included six articles with a sole focus on intraoperative exposure and subsequent hazards to operating staff.

**Table 1:** Review articles All systematic reviews encompassing intraoperative fluoroscopy exposure, delineated by specialty, and information category included in the review. Evidence level not included as articles were review articles

Title	Authors	Review focus	Categories
Ionising radiation and orthopaedics	Dewey et al., 2005 [1]	Orthopedic surgery	Radiation exposure, cancer prevalence/risk, radiation reduction, surgeon awareness/adherence
Hazards of ionizing radiation and its impact on spine surgery	Hadelsberg et al., 2016 [3]	Spine surgery	Radiation exposure, cancer prevalence/risk
Radiation exposure and health risks for the orthopaedic surgeon	Hayda et al., 2018 [4]	Orthopedic surgery	Radiation exposure, cancer prevalence/risk, radiation reduction, surgeon awareness/adherence
Radiation exposure in endovascular repair of abdominal and thoracic aortic aneurysms	Monastiriotis et al., 2015 [6]	Endovascular procedures	Radiation exposure, cancer prevalence/risk
What we know about intraoperative radiation exposure and hazards to operating theatre staff: a systematic review	Gogos et al., 2022 [7]	All fields	Radiation exposure, cancer prevalence/risk
Occupational hazards to the joint replacement surgeon: radiation exposure	Daryoush et al., 2021 [8]	Total joint arthroscopy/orthopedics	Radiation exposure, cancer prevalence/risk, radiation reduction
Is everyone covered? A resident’s perspective on radiation exposure in orthopedic surgery	Williamson et al., 2013 [13]	Orthopedic surgery	Radiation exposure, radiation reduction
Does less invasive spine surgery result in increased radiation exposure? A systematic review	Yu and Khan, 2014 [14]	Spine surgery	Radiation exposure, risk reduction, surgeon awareness/adherence
Ionizing radiation in endovascular interventions	Walsh et al. 2008 [15]	Endovascular procedures	Radiation exposure, risk reduction
The use of ionising radiation in orthopaedic surgery: principles, regulations and managing risk to surgeons and patients	Raza et al., 2021 [16]	Orthopedic surgery	Radiation exposure, cancer prevalence/risk, radiation reduction, surgeon awareness/adherence
Understanding the basis of radiation protection for endovascular procedures: occupational and patients	Rial and Vañó, 2021 [27]	Endovascular procedures	Radiation exposure, risk reduction
Radiation in the orthopedic operating room: what we know, what we do, and what needs attention	Hamid et al., 2025 [28]	Orthopedic surgery	Radiation exposure, cancer prevalence/risk, radiation reduction, surgeon awareness/adherence
Systematic review on radiation-induced DNA damage and cancer risk in endovascular operators	Maris et al., 2025 [29]	Vascular surgery	Cancer prevalence/risk, radiation exposure
Scoping review of robotics technology in spinal surgery with highlights of the Annual Seattle Science Foundation Course	Dietz et al., 2025 [30]	Orthopedic surgery	Radiation reduction techniques (other than shielding)
Imaging derived holograms improve surgical outcome in inexperienced surgeons: a meta-analysis	Demeco et al., 2025 [31]	All fields	Radiation reduction techniques (Other than shielding)

Scope of Accreditation

The American College of Radiology (ACR) accredits organizations that ensure that medical imaging staff have appropriate qualifications and that imaging modalities have met quality and safety requirements [32]. We utilized the “Find an ACR-Accredited Imaging Facility” tool to identify that all United States facilities were accredited.

Radiation exposure

Thirteen articles investigated intraoperative radiation exposure from fluoroscopy (Table 2). Most of them focused on radiation exposure to different body areas (58.3%, 7/13) [5,11,17,18,33-36], with the remaining articles stratifying radiation exposure by procedure type (3/13, 23.1%) [37-39], surgeon specialty (1/13, 7.7%) [40], and patient factors that affect exposure (1/13, 7.7%) [19].

**Table 2:** Articles on radiation exposure ^*^Denotes ACR-accredited institution A succinct summary of all identified articles discussing radiation exposure, further delineated by subgrouping and evidence level UOQ: upper outer quadrant; LIQ: lower inner quadrant; ACR: The American College of Radiology

Title	Authors	Subgrouping	Findings	Evidence level
Radiation hazards to vascular surgeon and scrub nurse in mobile fluoroscopy equipped hybrid vascular room	Kim et al., 2017 [5]	Area of exposure - body	Increased procedure complexity increases radiation exposure. The mortality risk of a vascular surgeon is 1 in 76 persons with exposure of 7.7 mSv per year	II
Radiation exposure to a pregnant urological surgeon - what is safe?	Birnie et al., 2015 [11]	Area of exposure - abdomen	Average doses to the abdomen were greater in the surgeon’s home than intraoperatively	III
Breast radiation exposure in female orthopaedic surgeons	Valone et al., 2016 [17]^*^	Area of exposure - breast	UOQ of the breast had significantly higher radiation exposure compared to the LIQ. The lateral C-arm position has higher radiation exposure for both areas compared to the standard position	II
Occupational radiation exposure in doctors: an analysis of exposure rates over 25 years	Sharkey et al., 2021 [18]	Area of exposure - body	Surgeons had significant decreases in radiation exposure to the body, hand, and neck; however, an increase to the eye over a 25-year span	III
Cumulative radiation exposure to patients undergoing arthroscopic hip preservation surgery and occupational radiation exposure to the surgical team	Canham et al., 2015 [19]^*^	Patient risk factors	Higher patient BMI correlated with a 10-fold and 25-fold increased risk of radiation exposure at 3 and 6 ft away from the source. Occupational exposure levels lead to a 0.001% risk of fatal cancer based on 4-month exposure	II
Occupational radiation exposure from C arm fluoroscopy during common orthopaedic surgical procedures and its prevention	Mahajan et al., 2015 [33]	Area of exposure - body	The dominant hand with the greatest radiation exposure. Mean operative time and mean exposure time are directly related. Closed reductions had higher exposure than open reductions	II
A report on occupational ionizing radiation exposure by an orthopedic surgeon in a national health-care setting - clinical case perspective	Abosala, 2018 [34]	Area of exposure - body	Varied radiation exposure in different procedures. The surgeon’s hands are most exposed to radiation, but are below the yearly limits based on a single surgeon's case exposure over 4 months	II
Trends in the use of radiation protection and radiation exposure of European endourologists: a prospective trial from the EULIS-YAU Endourology Group	Fontanet Soler et al., 2024 [35]	Area of exposure - body	100% and 90% of surgeons surveyed use thyroid shields and lead aprons, 60% wear leaded glasses, and 90% use dosimeters under the aprons	V
Insights into testicular radiation exposure in the orthopedic surgeon	Zak et al., 2025 [36]	Area of exposure - gonads	Without lead protection, a surgeon would reach the annual limit within 100 hours of operating. The use of an apron on an anthropomorphic model resulted in significantly less radiation exposure to the testicular area (p<0.001)	II
Surgeon and staff radiation exposure during radioguided parathyroidectomy at a high volume institution	Oltmann et al., 2014 [37]^*^	Procedure type - head and neck	A surgeon needs radiation education after 30 cases and needs monitoring after 150 cases of radio-guided parathyroidectomy	II
Radiation exposure to the surgeon during fluoroscopically assisted percutaneous vertebroplasty: a prospective study	Harstall et al., 2005 [38]	Procedure type - spine	Vertebroplasty has high levels of radiation time, increasing the dose to the surgeon. Thyroid and vest shielding significantly reduces dose exposure. Based on 3 consecutive months	II
Radiation exposure to the surgeon and the patient during kyphoplasty	Mroz et al., 2008 [39]^*^	Procedure type - spine	Exposure dose to the surgeon directly correlates with exposure time. Based on average exposure from 27 kyphoplasties, the surgeon's hands would exceed the yearly allowance after 300 cases	II
Radiation exposure and case characteristics in national sample of female orthopaedic trauma and arthroplasty surgeons	Lai et al., 2020 [40]^*^	Surgeon specialty	Trauma surgeons are exposed to greater radiation compared to arthroplasty surgeons	II

Areas of Exposure

In a prospective study involving 12 surgeons, Mahajan et al. reported that the surgeon’s dominant hand experienced the highest radiation exposure (2.92 mSv/year), followed by the non-dominant hand (2.32 mSv/year), neck (1.312 mSv/year), chest (0.68 mSv/year), and gonads (0.60 mSv/year) [33]. Similarly, a study conducted by Abosala reported that the surgeon’s finger received the highest intraoperative radiation dose, estimated at 3.5 mSv/year [34]. Fontanet Soler et al.'s multicenter study across four years also found that the surgeon’s hands received the greatest dose (11 mSv/year), followed by eyes and chest (1.65 and 1.35 mSv/year) [35]. A retrospective study by Sharkey et al. observed that while hand and thyroid exposure have remained consistent over 25 years, eye exposure progressively increased [18].

Kim et al. were the first to perform a prospective study investigating composite total body radiation exposure, finding that vascular surgeons had 7.70 mSv/yr, while scrub nurses had 2.62 mSv/yr, wearing lead goggles, a thyroid shield, and a lead apron. Notably, surgeons with higher productivity levels could feasibly reach the 20 mSv/year exposure limits despite shielding [5]. Zak et al. demonstrated this with an anthropomorphic model, showing that a male surgeon would reach the ICRP annual dose limit to the gonads within 100 hours of operating without utilizing lead shielding; however, with protection, this exposure was significantly decreased (p<0.001) [36].

Valone et al. conducted the only study examining breast radiation exposure, using an anthropomorphic torso model to show that the upper outer quadrant (UOQ) of the breast remains susceptible to intraoperative radiation despite the use of a lead apron, with exposure measured at 0.004 mSv/hour (0.4 mrem/hour) [17]. The amount of radiation was found to increase if the vest did not properly fit the surgeon [17]. Likewise, only one study has examined radiation exposure to the lower abdomen. In a case study, Birnie et al. reported that the abdomen received higher radiation at home (0.12 µSv/hour) compared to the operating room (0.065 µSv/hour) with shielding utilization [11].

Procedure Type

Oltmann et al. found that surgeons performing radio-guided parathyroidectomies could complete up to 5,625 cases before reaching the annual maximum permissible deep dose of 45 mSv (4,500 mrem), as defined by U.S. NRC guidelines [21,37]. They also noted that the scrubbed operating room nurse and anesthesiologist would require nearly two and four times, respectively, the number of cases to reach the same exposure limit [37]. The remainder of the articles focused on spine-specific cases. A prospective study by Harstall et al. found that radiation doses to the thyroid, left hand, right hand, and left arm of a surgeon wearing only a lead apron performing vertebroplasty exceeded the yearly limit at 31.2mSv, 63.7 mSv, 29.4 mSv, and 50 mSv, respectively [38]. Mroz et al. published a similar study related to kyphoplasty cases, with surgeons wearing thyroid shields and lead aprons. Notably, the only dosimeters that returned values above the minimum reportable dose were the dosimeters on the outside of the lead protection, with the hands receiving the highest dosage at 0.413 mSv/min [39]. Based on the amount of exposure, the researchers recommended a case load limit of 300 per year [39].

Surgeon Specialty

Lai et al. compared radiation exposure between orthopedic trauma surgeons and arthroplasty surgeons. In their study of 40 female surgeons, trauma surgeons used fluoroscopy more frequently (213.54 seconds/day vs. 25.21 seconds/day, p<0.001) and had correspondingly higher outside dosimeter readings after use for 10 days (0.766 mSv (76.60 mrem) vs. 0.05 mSv (5.0 mrem, p=0.01) [40]. This is in concordance with Mahajan et al.’s finding of a positive correlation between operative time and exposure time [33]. Of note, this only included time in the operating room, not trauma surgeons’ exposure while utilizing CT trauma gram.

Patient Factors

Canham et al. reported that higher patient BMI was associated with increased radiation exposure to operating room personnel [19]. This finding was limited to arthroscopic hip preservation surgery and has not yet been demonstrated in other procedures or surgical specialties.

Radiation shielding

Among the nine articles addressing radiation shielding, 33.3% (3/9) [2,12,26] focused on thyroid protection, 25% (2/9) [41,42] highlighted the advantages of axillary shielding, 33.3% (3/9) [43-45] examined the effectiveness of protective eyewear, and 12.5% (1/8) discussed the use of lead surgical caps (Table 3) [46].

**Table 3:** Articles on radiation shielding ^*^Denotes ACR-accredited institution All identified articles discussing the benefit of lead shielding, organized based on shielding type UOQ: upper outer quadrant; LIQ: lower inner quadrant; ACR: The American College of Radiology

Title	Authors	Shield type	Finding	Evidence level
The need to protect the thyroid gland during image intensifier use in orthopaedic procedures	Devalia et al., 2004 [2]	Thyroid	Prospective study of 44 cases. Nearly 97% in the operating room were wearing lead vests, but only 4% wore thyroid shields	II
Radiation exposure during fluoroscopy: should we be protecting our thyroids?	Tse et al., 1999 [12]	Thyroid	23 times reduction in thyroid radiation exposure when a shield was utilized across 20 procedures in 6 weeks	II
Evaluation of thyroid shields for reduction of radiation exposure to orthopaedic surgeons	Dewey et al., 2008 [26]	Thyroid	Thyroid shielding reduced radiation exposure in one trainee by a factor of 13. 54% of trainees utilized shields due to their availability. Radiation exposure to the thyroid of 19 orthopedic trainees over a period of 3 months	II
Methods for reducing intraoperative breast radiation exposure of orthopedic surgeons	Van Nortwicket al., 2021 [41]^*^	Axillary	Axillary shields decreased the radiation exposure of the UOQ of breasts in an anthropomorphic torso model	II
The addition of a leaded arm sleeve to leaded aprons further decreases operator UOQ chest wall dose during fluoroscopically guided interventions	Ramanan et al., 2024 [42]^*^	Axillary	Prospective cohort of over 61 fluoroscopically guided interventions. The addition of a leaded sleeve decreases radiation attenuation of the UOQ of the breast and the axillary region by 34% vs. a lead apron alone (96% vs. 62%)	II
Leaded eyeglasses substantially reduce radiation exposure of the surgeon's eyes during acquisition of typical fluoroscopic views of the hip and pelvis	Burns et al., 2013 [43]^*^	Glasses	Use of leaded glasses reduced the radiation dose to the eye by a factor of 10. Surgeon phantoms were utilized for 16 different positions for the hip and pelvis	II
Evaluation of factors associated with the effectiveness of radiation protection glasses	Imai et al., 2023 [44]	Glasses	A reater lead equivalent of protective glasses leads to greater protection from radiation	III
Evaluation of factors influencing eye lens radiation dose while using radiation protection glasses in interventional radiology: a phantom study	Imai et al., 2025 [45]	Glasses	Increasing lead equivalent dose decreases lead radiation dose when an anthropomorphic model face was oriented at 0 (-0.944) or 45 degrees (-0.963)	III
Radiation brain dose to vascular surgeons during fluoroscopically guided interventions is not effectively reduced by wearing lead equivalent surgical caps	Kirkwood et al., [46]^*^	Lead cap	Prospective study at a single institution, involving 29 procedures. Radiation attenuation of the middle brain is not effectively decreased by a radiation cap	II

Thyroid Shields

In 1998, Dewey et al. demonstrated a reduction in thyroid radiation exposure by a factor of 13, citing one of the trainees in this study who wore a dosimeter [26]. Despite this, only 37% of surgeon trainees opted to wear thyroid shielding during fluoroscopic procedures [26]. A year later, Tse et al. demonstrated a reduction in thyroid radiation exposure by 23-fold with the use of a thyroid shield [12]. Five years later, Devalia et al. demonstrated that without shielding, 44 routine orthopedic surgery procedures, such as intramedullary nailing, exposed healthcare providers to over 4,357.5 mSv/cm² (4,357.5 mGy/cm²), far exceeding the 1 Sv level associated with an increased risk of thyroid carcinoma [2].

Axillary shields

Van Nortwick et al. performed the first study quantifying axillary radiation reduction via axillary shielding. Their findings showed that lead sleeves and axillary supplements decreased intraoperative radiation exposure to the UOQ of the breast compared to a lead vest alone (0.013 mSv/hr (1.3 mrem/hr) vs. 0.594 mSv/hr (59.4 mrem/hr) vs. 0.974 mSv/hr (97.4 mrem/hr, p<0.01) on an anthropomorphic model [41]. Ramanan et al. were the first and only group to demonstrate these findings in a clinical setting among vascular surgeons, showing that lead sleeve protection reduced radiation exposure to the UOQ of the chest wall by 96% (p<0.001) [42].

Protective Glasses

Burns et al. and Imai et al. showed that protective glasses reduced eye radiation by a factor of ten and six, respectively [43,44]. The most effective glasses were those that fit snugly (with no gap between the model “skin” and the lenses), had the highest lead equivalence (0.75 mmPb), and offered a surface area of 36.9 cm² [44,45]. Radiation to the eye, specifically the posterior subcapsular region, has been linked to cataract development [43]. Currently, no safe exposure threshold has been established for the lens of the eye [43].

Surgical Cap

In a prospective study, Kirkwood et al. evaluated the efficacy of radiation caps in decreasing brain radiation [46]. While the cap was shown to decrease skull radiation during 29 endovascular procedures, no reduction was observed in the middle brain, and it was noted that the skull itself can block approximately 40% of scatter radiation [46].

Radiation reduction techniques (other than shielding)

Of the 13 articles on radiation reduction techniques (Table 4), 38.5% (5/13) [47-51] focused on surgical approaches, 15.4% (2/13) [52,53] on C-arm positioning, 15.4% (2/13) [54,55] on how surgeon experience is protective of radiation exposure, 15.4% (2/13) [56,57] on physician awareness of radiation exposure, 7.7% (1/13) [58] on protective surgeon positioning, and 7.7% (1/13) [59] on equipment’s impact on exposure.

**Table 4:** Articles on radiation reduction techniques (other than shielding) *Denotes ACR-accredited institution A brief summary of the articles discussing intraoperative radiation reduction techniques that do not involve lead shielding ACR: The American College of Radiology

Title	Authors	Reduction technique	Finding	Evidence level
The Surgical Approach Visualization and Navigation (SAVN) system reduces radiation dosage and surgical trauma due to accurate intraoperative guidance	Jiang et al., 2019 [47]	Surgical approach	Use of SAVN reduced intraoperative radiation by 36.2% in 20 patients	II
Navigation versus fluoroscopy in multilevel MIS pedicle screw insertion: separate analysis of exposure to radiation of the surgeon and of the patients	Konieczny and Krauspe 2019 [48]	Surgical approach	Percutaneous pedicle screw insertion guided by navigation reduces radiation exposure by 80% compared to insertion by fluoroscopy	II
Sensorless based haptic feedback integration in robot-assisted pedicle screw insertion for lumbar spine surgery: a preliminary cadaveric study	Nakdhamabhorn et al., 2024 [49]	Surgical approach	A hospital in Thailand developed a robotic system with sensorless haptic feedback that reduces radiation by minimizing fluoroscopy use. It enables remote, accurate pedicle screw insertion with minimal errors (as low as 0.011 rad and 0.0057 Nm), outperforming traditional techniques	II
Occupational radiation dose during transcatheter aortic valve implantation (TAVI)	Sauren et al., 2011 [50]	Surgical approach	Transapical TAVI procedures expose the surgeon to higher levels of radiation compared to a transfemoral approach. Transapical doses limit the surgeon to 250 of this procedure/year before receiving the maximum recommended dose	II
The effect of a simple and reproducible marking technique on enhancing radiation safety in surgical fixation of proximal femur fractures	Khan et al., 2025 [51]	Surgical approach	A case control study with 125 patients at a general hospital in the UK, utilizing a marking technique, shows a mean DAP decrease by 45% (p=0.0001) and a 27% decrease in exposure time	II
Minimizing surgeon radiation exposure during operative treatment of pediatric supracondylar humerus fractures	Montgomery et al., 2023 [52]*	Equipment position	C-arm position in the inverted position exposed the surgeon to up to 7.8% more effective dose of radiation	II
Investigation of radiation dose around C-arm fluoroscopy and relevant cancer risk to operating room staff	Robatjazi et al., 2022 [53]	Equipment position	The optimal positioning for a surgeon to receive the least radiation exposure should be 60 degrees from the image intensifier. 30 and 330 degrees from the image intensifier have the highest radiation dosage	III
Does surgical experience decrease radiation exposure in the operating room?	Magee et al., 2021 [54]*	Physician experience	Retrospective cohort study. Greater physician experience led to a reduction in fluoroscopy time and effective dose exposure. 10+ years of experience led to a 43% decrease in DAP compared to <1 year of experience	III
Level of supervision and radiation exposure of senior orthopedic residents during surgical treatment of proximal femur fracture	Carrazana-Suarez et al., 2021 [55]*	Physician experience	Level of supervision did not have a statistically significant reduction in fluoroscopy time or radiation exposure in 23 patients	III
The effect of an intraoperative real-time counter on radiation exposure events during operative treatment of distal radius fractures	Delma et al., 2023 [56]*	Awareness of exposure amount	Real-time dosimeter decreased radiation dose by 37.5%, radiation exposure time by 42%, and operative time by 13.5% across 160 patients	II
Real-time dosimetry reduces radiation exposure of orthopaedic surgeons	Müller et al., 2014 [57]	Awareness of exposure amount	Real-time dosimeter reduced fluoroscopy time by up to 42% and radiation dose by 45% across 68 patients	III
Radiation exposure during scoliosis surgery: a prospective study	McArthur et al., 2015 [58]	Physician position	Surgeon radiation dose decreased by 74% when positioned on the opposite side of the imaging source in 30 patients	II
A study of occupational radiation dosimetry during fluoroscopically guided simulated urological surgery in the lithotomy position	Horsburgh et al., 2016 [59]	Multiple strategies	Use of pulsed fluoroscopy reduced surgeon dose by 67% to 77% across the body, eyes, and legs. Sitting significantly increased the surgeon’s exposure by 78%. Increasing distance from the patient to the imaging source reduced surgeon exposure by 5-25%. Lead protection mounted on the table reduced exposure by 34-38%. The conjunction of all protective measures resulted in an average whole-body reduction by 97%	II

Equipment/Approach

Jiang et al. compared surgeon radiation exposure in thoracic spinal tumor resection using standard C-arm position versus the Surgical Approach Visualization and Navigation (SAVN) system. The SAVN approach reduced total radiation exposure to the head and neck region by 72.1%, due to the decreased image frames needed to obtain an acceptable image, and a 36.2% decrease in operative time [47]. Konieczny and Krauspe also investigated radiation reduction via navigation during percutaneous pedicle screw placement. This group identified an 80% reduction in radiation exposure compared to fluoroscopy (13.3 vs. 66.8 cGyxcm^2^, p<0.01), highlighting the beneficial effect of emerging technology on occupational safety [48]. Similarly, Nakdhamabhorn et al. demonstrated a remote, robotic system that eliminated surgeon radiation exposure beyond setup and final images. This system outperformed traditional pedicle screw insertion techniques with minimal errors [49].

Sauren et al. compared transapical vs. transfemoral approaches for transcatheter aortic valve placement. The cardiothoracic surgeon received a higher average effective radiation dose by a factor of 10 when utilizing the transapical approach (0.03 vs. 0.003 mSv, p<0.05) and had a higher intraoperative fluoroscopy time (18 vs. 6 mins, p<0.05), correlating with the navigational spine teams’ findings of increased fluoroscopy time increasing radiation exposure [50]. Khan et al. utilized a preoperation marking technique to decrease fluoroscopy exposure dose area product (DAP) by 45 % (p=0.0001) and usage time by 27% (p=0.001). They used a permanent marker to mark the original laser position on the AP and lateral views to guide subsequent images. This simple technique was highly effective in reducing both time and dose [51].

Equipment Position

Montgomery et al. utilized a simulated operating room to study how C-arm positioning affects the surgeon during pediatric supracondylar humerus fractures. The inverted C-arm exposed the surgeon to an average effective dose increase of 6.6% of radiation exposure compared to the standard position [52]. Robatjazi et al. also investigated C-arm positioning, utilizing a water tank as a radiation scatter medium to evaluate how different angles, heights, and distances from the scatter source impact surgeon radiation exposure. They determined that positions behind the X-ray tube and image intensifier resulted in the lowest radiation exposure, though they acknowledged these orientations were not clinically practical [53]. Practical positions that had the least amount of radiation exposure were located at 60 degrees in either direction from the image intensifier, and the position that had the greatest amount of radiation exposure was at 30 degrees in either direction from the intensifier [53].

Physician Experience

In a retrospective cohort study of 759 patients and 17 attending surgeons, Magee et al. found that surgeons with 10+ years of experience had decreased fluoroscopy usage, including time in seconds (42.1 vs. 56.9, p=0.00011), number of images (57.6 vs. 74.5, p=0.0008), and radiation dose (110.4 mSv vs. 179.9 mSv, p=0.001) compared to surgeons with less than one year of experience [54]. Comparatively, Carrazana-Suarez et al. found that the level of senior resident supervision had no effect on reducing intraoperative time during 23 proximal femur fractures [55].

Awareness of the Amount of Radiation Exposure

Real-time dosimeters have been shown to reduce radiation exposure by increasing intraoperative surgeon awareness during fluoroscopic procedures [56]. Delma et al. demonstrated a significant reduction in the amount of radiation exposure events (77.2 vs. 39.9; p<0.05), time (41.9 vs. 24.2 seconds, p<0.05), and dose (0.8 vs. 0.5 mSv, p<0.05) when using intraoperative radiographic counters for 135 distal radius fractures [56]. Similarly, Müller et al. reported decreased radiation time and dose for a variety of fracture patterns (p<0.05) [57].

Physician Position

Surgeons can reduce radiation exposure via increased distance from the X-ray source. McArthur et al. demonstrated that radiation exposure was significantly decreased when the surgeons in scoliosis procedures across six months positioned themselves on the opposite side of the X-ray emitter (62 uSv vs. 16 uSv) [58]. Horsburgh et al. found that sitting to operate with fluoroscopy gave the surgeon 17% higher radiation dose compared to standing, with a 78% higher external dose to the genitalia [59]. Table 5 provides a summary of the articles with illustrations for proper surgeon, equipment, and other personnel positioning, as well as scatter radiation exposure.

**Table 5:** Articles with positioning illustration ^*^Denotes ACR-accredited institution A table depicting the articles with relevant positioning and radiation exposure illustrations ACR: The American College of Radiology

Illustration type	Title	Authors
Surgeon positioning, equipment positioning, scatter radiation exposure	Ionising radiation and orthopaedics	Dewey et al., 2005 [1]
Equipment positioning	Occupational hazards to the joint replacement surgeon: radiation exposure	Daryoush et al., 2021 [8]
Surgeon positioning, equipment positioning, other personnel positioning, scatter radiation exposure	Does less invasive spine surgery result in increased radiation exposure? A systematic review	Yu et al., 2014 [14]
Equipment positioning	The use of ionising radiation in orthopaedic surgery: principles, regulations and managing risk to surgeons and patients	Raza et al., 2021 [16]
Surgeon positioning, equipment positioning	Breast radiation exposure in female orthopaedic surgeons	Valone et al., 2016 [17]^*^
Scatter radiation exposure	Cumulative radiation exposure to patients undergoing arthroscopic hip preservation surgery and occupational radiation exposure to the surgical team	Canham et al., 2015 [19]^*^
Scatter radiation exposure	Occupational radiation exposure from C arm fluoroscopy during common orthopaedic surgical procedures and its prevention	Mahajan et al., 2015 [33]
Surgeon positioning, equipment positioning	Methods for reducing intraoperative breast radiation exposure of orthopedic surgeons	Van Nortwick et al., 2021 [41]^*^
Surgeon positioning	Leaded eyeglasses substantially reduce radiation exposure of the surgeon's eyes during acquisition of typical fluoroscopic views of the hip and pelvis	Burns et al., 2013 [43]^*^
Surgeon positioning, other personnel positioning	Occupational radiation dose during transcatheter aortic valve implantation	Sauren et al., 2011 [50]
Equipment positioning	Are Indian orthopaedic surgeons aware of the health hazards of radiation exposure? A survey and review on awareness and ways to mitigate them	Sheth et al., 2022 [60]

Cancer prevalence and risk

Twelve articles investigated cancer prevalence and risk (Table 6), organized by cancer/risk type: 25% (3/12) [61-63] on all cancer prevalence, 16.7% (2/12) on breast cancer prevalence [64,65], 16.7% (2/12) offspring/fetal risk [9,10], 8.3% [66] cutaneous small cell carcinoma, 8.3% [67] on cataracts, 16.7% [68,69] on DNA damage markers in operators, and 8.3% [70] on female fertility impact.

**Table 6:** Articles on cancer prevalence/risk ^*^Denotes ACR-accredited institution A table depicting the known cancer prevalence and risk attributed to intraoperative fluoroscopy exposure SCC: squamous cell carcinoma' OR: odds ratio; CI: confidence interval; ACR: The American College of Radiology

Title	Authors	Risk type	Finding	Evidence level
Image-guided reconstruction of femoral fractures: is the staff progeny safe?	Theocharopoulos et al., 2005 [9]	Offspring	Dose to the abdomen heavily depends on the use of PPE and the positioning of the surgeon. Moving from the patient's affected side to the contralateral position significantly reduces abdominal exposure. Based on permissible fetal doses, the use of lead properly protects the intrauterine fetus from excessive risk	II
Ionising radiation: are orthopaedic surgeons' offspring at risk?	Zadeh and Briggs 1997 [10]	Offspring	Congenital abnormalities were significantly higher in orthopedic surgeons and gynecologists, leading to the belief that exposure to radiation is not the likely cause of birth defects in children of surgeons	III
Increased cancer risk among surgeons in an orthopaedic hospital	Mastrangelo et al., 2005 [61]	All cancer	Surgeons with a higher risk of tumors vs. controls	II
Cancer prevalence among a cross-sectional survey of female orthopedic, urology, and plastic surgeons in the United States	Chou et al., 2015 [62]^*^	All cancer	Female orthopedic surgeons had a higher prevalence of all-cause (1.9) and breast cancer (2.9) compared to the general population. Urology (54%) and orthopedics (37%) reported commonly using fluoroscopy	III
Increased breast cancer prevalence among female orthopedic surgeons	Chou et al., 2012 [63]^*^	All cancer	The prevalence of cancer was 189% higher in female orthopedic surgeons compared with the general U.S. female population. 1.89-fold higher prevalence of all-cause cancer and 3.97-fold higher prevalence of breast cancer in female orthopedic surgeons	III
Increased prevalence of breast and all-cause cancer in female orthopaedic surgeons	Chou et al., 2022 [64]^*^	Breast cancer	Female orthopedic surgeons had an 85% higher prevalence of breast cancer compared to the general US population	III
Occupational exposure to ionizing radiation in female physicians and breast cancer risk: a systematic review and meta-analysis	Cristófalo et al., 2025 [65]	Breast cancer	Search of 34,744 participants; found that increased breast cancer risk among female physicians exposed to ionizing radiation, OR 1.84 (CI 1.11-3.06). Female physicians exposed to ionizing radiation have a higher risk of breast cancer compared to those who are not exposed	III
Squamous cell carcinoma on the fingers of orthopedic surgeon induced by occupational radiation exposure	Yoon et al., 2019 [66]	Cutaneous SCC	Case report of a 49-year-old orthopedic surgeon. High volume with no radiation protection on the hands. Presented with necrotic lesions on the hands and diagnosed with SCC due to occupational radiation exposure	V
Prevalence of cataractous changes in the eyes and chronic inflammatory changes in the hands among spine surgeons	Hijikata et al., 2025 [67]	Cataracts	In a cross-sectional study of 162 orthopedic/spine surgeons in Japan, 38% had chronic hand inflammation, significantly associated with hand radiation exposure (p<0.001). Use of a navigation system showed no significant effect (p=0.63). Cataracts were present in 20% of surgeons, with another 20% showing early cataractous changes	III
Radiation-induced DNA damage in operators performing endovascular aortic repair	El-Sayed et al., 2017 [68]	DNA damage	Increased levels of DNA damage markers were found in the lymph nodes of operators after performing endovascular aortic repair with fluoroscopy. Utilization of lead protection completely reduced the levels of the DNA damage marker	I
Radiation-induced miRNAs changes and cf mtDNA level in trauma surgeons: epigenetic and molecular biomarkers of X-ray exposure	Kussainova et al., 2024 [69]	DNA damage	Mutant DNA that was obtained in blood samples from 30 trauma surgeons was more than 4 times the level of 56 individuals who were not exposed to fluoroscopy (P<0.001). Stratified for smoking and still had nearly three times the mutant DNA compared to their smoking non-exposed counterparts (p=0.01)	III
Health considerations for female orthopaedic surgeons	Chou et al., 2023 [70]^*^	Female fertility	Female orthopedic surgeons have higher rates of infertility and complications compared to the U.S. general population	V

All Cancer

In a 2005 retrospective cohort study, the first publication to link orthopedic surgeons with an elevated cancer risk reported that, based on logistic regression analysis of 158 workers undergoing routine dose assessments, employment as an orthopedic surgeon was associated with a significantly higher likelihood of developing cancer (p<0.002) [61]. Chou et al. additionally surveyed female surgeons in urology, plastic surgery, and orthopedic surgery to assess fluoroscopic exposure and cancer risk [62,63]. Only orthopedic surgeons were found to have a significantly greater prevalence of any cancer (standardized prevalence ratio (SPR) 1.85; 95% CI: 1.19-2.76) and breast cancer (SPR: 2.90; 95% CI: 1.66-4.71), despite more urologists (54%) reporting multi-weekly fluoroscopy usage than orthopedists (31%) [62,63].

Breast Cancer

Chou et al. expanded upon their initial study in 2022 by surveying 672 female orthopedic surgeons and found a 1.89-fold higher prevalence of all-cause cancer and 3.97-fold higher prevalence of breast cancer in this population when compared to the general female population of the United States [64]. Cristófalo et al. similarly found among 34,744 participants that females exposed to ionizing radiation had an odds ratio of 1.84 of a higher risk of having breast cancer [65].

Cutaneous Squamous Cell Carcinoma

Yoo et al. reported a case of a 49-year-old male who presented with necrotic squamous cell carcinoma on his hands. The patient self-reported using fluoroscopy without gloves for 11 years for 700 percutaneous vertebral augmentations [66].

Cataracts

In a cross-sectional study of 162 orthopedic/spine surgeons in Japan, Hijikata et al. found that 20% of surgeons had cataracts present, and another 20% had early cataract changes present [67].

DNA Damage

El-Sayed et al. measured markers of DNA damage in the lymphocytes of endovascular surgeons before, immediately after, and 24 hours after utilizing fluoroscopy with and without leg shielding in their procedures. Levels of DNA damage markers immediately after procedures were found to increase significantly in the surgeon’s lymphocytes (p<0.0003) [68]. When operators wore leg shielding and then had the same blood sampling, DNA damage markers were not found in the lymphocytes of the operators, indicating the protective nature of the shielding [68]. Kussainova et al. concurred with these findings, finding that trauma surgeons exposed to fluoroscopy had higher levels of DNA damage markers in their blood compared to controls. They also found a dose-dependent relationship between fluoroscopy exposure and the level of damaged DNA markers [69].

Offspring

Zadeh et al. surveyed orthopedists and obstetricians as a control group to investigate an association between congenital abnormalities and fluoroscopy exposure. While there was a significant difference in the rate of congenital abnormalities for both orthopedists and obstetricians compared to the general population (p<0.001), there was no significant difference between physicians exposed to intraoperative radiation vs. not (p=0.78) [10]. Furthermore, Theocharopoulos et al. demonstrated on an anthropomorphic model that the use of lead protection adequately protects an intrauterine fetus from excessive risk during fluoroscopic procedures, regardless of surgeon positioning. No studies to date have identified risk to surgeon offspring if appropriate shielding is worn during fluoroscopic cases [9]. Chou et al. discussed the impact of orthopedic surgery on the fertility of female orthopedic surgeons, finding a 17% higher infertility rate among female orthopedic surgeons compared to the U.S. general population [70]. It should be noted that the amount of radiation exposure in these surgeons was not studied.

Radiation safety awareness and adherence

All nine of the articles discussing surgeon awareness and adherence to radiation safety protocols (Table 7) were survey-based. Sheth et al. demonstrated that 50% of Indian orthopedists in their study population were unaware of the appropriate 2-meter distance needed between the radiation source and surgeon to minimize radiation exposure [60]. Similarly, 66.7% of orthopedists in Jordan, and 61% of United States orthopedic residents were unaware of this safety guideline [24,23]. Only 18% and 10.8% of orthopedic surgeons in India and US orthopedic residents, respectively, were aware of yearly recommended allowable doses of radiation [23,60]. Together, these three studies highlight the low levels of radiation safety knowledge within the surgical community.

**Table 7:** Articles on guideline awareness and adherence *Denotes ACR-accredited institution This table summarizes articles on the importance of surgeon awareness and adherence to radiation safety guidelines PPE: personal protective equipment; ACR: The American College of Radiology

Title	Authors	Findings	Evidence level
A preliminary survey of women orthopaedic surgeons on awareness of radiation safety practice and breast cancer risk in India	John and Madhuri, 2021 [22]	The weight of the shield has a negative effect on adherence. Weight caused 9.8% incompliance. 88.2% of surgeons surveyed were unaware of the amount of radiation received	V
Decreasing exposure to thyroid radiation in an orthopaedic theatre setting: an educational intervention	Duggan et al., 2023 [23]	Education about radiation practices increases adherence. Post-intervention had an 89.7% increased likelihood of use of a thyroid shield after education	V
Knowledge and practice of radiation protection in the operating theater among orthopedic surgeons	Al Mohammad et al., 2022 [24]	Surgeons with radiation training had an 8.6% higher score on the application of radiation knowledge compared to a control group without education	V
What leads to lead; results of a nationwide survey exploring attitudes and practices of orthopaedic surgery residents regarding radiation safety	Bowman et al., 2018 [25]^*^	Noncompliance related to the inability to locate supplies, providing individual shields associated with higher compliance	V
Are Indian orthopaedic surgeons aware of the health hazards of radiation exposure? A survey and review on awareness and ways to mitigate them	Sheth et al., 2022 [60]	Surgeons are unaware of the factors impacting radiation exposure and the proper safety guidelines	V
Intraoperative ionizing radiation exposure awareness and associated morbidity in neurosurgery: a nationwide survey	McCloskey et al., 2024 [71]	227 respondents from the American Association of Neurological Surgeons members. Assessed adherence with PPE thyroid (75%), lead apron (89%), and dosimeter (59%). Only 3% could correctly identify the safety limit for occupational radiation dose. Found that the likelihood of developing benign thyroid nodular disease (p=0.01) and leukemia (p=0.02)	V
Radiation exposure safety patterns with the use of intraoperative fluoroscopy	Kaplan et al., 2018 [72]	Use of a mini C-arm decreases compliance. Women were found to be more likely to be compliant with radiation safety practices. Surgeons with more recent education experiences were more likely to utilize radiation safety practices	V
A survey of UK standards of radiation protection amongst orthopaedic surgeons	Ninkovic-Hall et al., 2025 [73]	Low response rate (0.4% (consultants) and 5.3% (residents) of the UK workforce). Survey about knowledge of radiation and use of proper PPE	V
Radiation protection in the orthopedics department: insights from a cross-sectional study	Abanomy 2024 [74]	Cross-sectional study of 102 orthopedic surgeons in Saudi Arabia. Highlighted the gaps in radiation PPE use and knowledge, as well as only 3.9% provided formal education on radiation protection, 7.8%: basic understanding of ALARA, 80% did not use dosimeters	V

Additionally, these studies demonstrate widespread noncompliance with shielding - 83% of Indian orthopedists in Sheth et al.'s study never wore thyroid shields. In comparison, only 64% of Jordan orthopedists reported using personal protective equipment (PPE) most of the time [24]. The majority of neurological surgeons from the American Association of Neurological Surgeons cited compliance with PPE, both thyroid (75%) and lead apron (89%) [71]. Orthopedic surgery residents had the highest level of shielding compliance at 94% for aprons and 87% for thyroid shields [24]. In contrast, Kaplan et al. found that 42% of members of the American Society for Surgery of the Hand reported not wearing lead protection during mini-fluoroscopy procedures [72].

 Reasons for shielding noncompliance included low knowledge of radiation exposure [22,23,25], weight of equipment [22], and difficulty obtaining equipment [23,73]. Additionally, a lack of formal education on radiation safety was found in 96% of orthopedic surgeons surveyed in Saudi Arabia [74]. Duggan et al. found that after educational intervention, surgeons were more likely to adhere to thyroid shielding (pre: 11.3% vs. post: 46.2%, p=0.001) [23]. While the weight of equipment cannot be addressed, providing easier access to equipment is easily remedied. The researchers determined that for every additional protective unit made available, there was a 12.7% increase in usage in a group of surgeons, nurses, radiographers, anesthetists, and other operating room staff (p=0.009) [23].

Discussion

Given the risks associated with ionizing radiation, surgeons must be well-informed about radiation exposure, its potential health effects, and appropriate protective measures to safeguard themselves and operating room personnel [23,25]. In this paper, we present the first multispecialty review of occupational fluoroscopy exposure among surgeons, encompassing six key themes across 71 studies. Although urology and orthopedics are reported to have the highest rates of fluoroscopy use [64], all specialties employing this technology should be well-informed, as no level of radiation exposure is considered completely safe [11]. This review serves as a comprehensive reference for busy surgeons across all disciplines.

The most recent update on radiation safety information from the American Academy of Orthopaedic Surgeons, adopted in 2017, highlights the importance of shielding and the use of dosimeters [75]. Notably, while emphasizing the need for adopting local guidelines and decreasing overall X-ray use, this information statement stressed the need for the sites to “own” their radiation safety and lacked specific details of exposure limits or levels. Additionally, the Society of Interventional Radiology and the Cardiovascular and Interventional Radiology Society of Europe made a joint statement in May 2010 for radiation practice safety guidelines. These guidelines detail exposure limits, potential for exposure that surpasses these limits, and advice to decrease exposure [75,76]. Future research should explore the development of a joint standard protocol for radiation safety across specialties that would be externally applicable and the impact that this would have on safety protocol adherence.

Most of the literature (21.8%) focused on radiation exposure, specifically scatter radiation, and preventing this “indirect” exposure. However, in a multicenter review involving 10 endourologists, Magee et al. reported that in 35% of cases, the operator’s extremity appeared in at least one saved image when the surgeon had less than one year of experience [54]. This finding suggests that surgeons’ hands and arms are frequently positioned within the direct radiation field, substantially increasing the risk of radiation injury, especially to the extremities, which receive the highest radiation doses. [33]. In a specific case correlation, Yoon et al. covered a case report of an orthopedic surgeon with squamous cell carcinoma of the hands as a result of occupational radiation exposure [66]. Future research should include direct line radiation exposure and the necessity of a surgeon to have their extremity in the fluoroscopy frame.

The most effective radiation reduction technique is lead shielding, including vests and thyroid shields. Many studies [9,17,41,54] utilized anthropomorphic models to investigate radiation exposure and the effectiveness of lead shielding to protect from future cancer risk. Surgeons are responsible for overseeing both radiation exposure levels and the implementation of protective measures for operating room staff. Although staff members typically receive lower radiation doses than surgeons [5,7,24,37,53], it is important to emphasize that no level of radiation exposure is considered completely safe [20,21]. Many studies have primarily focused on surgeons as the main intervention group, treating staff as a secondary consideration. This approach may distort findings related to staff compliance, as variables less relevant to surgeons - such as high personnel turnover and the challenge of delivering consistent educational interventions across all shifts - can affect the reliability of results [37].

Limitations

Due to the heterogeneity in study design, a formal meta-analysis was not feasible. Instead, this review summarizes the frequency of reported shielding practices and radiation exposure metrics using descriptive statistics. Additionally, this paper includes studies that use anthropomorphic torsos to model the fit of lead protection. Although a useful tool, anthropomorphic models do not account for human variation in body type and resultant fit, which is crucial. This is best demonstrated in the eyeglasses model, where the space between the skin and eyewear allows scatter radiation to attenuate around the shielding [44]. Furthermore, the anthropomorphic models used to study axillary shielding do not have arms, which could be protective of the axilla and UOQ of the breast [41]. Lastly, with this model type, surgeon movement is not considered. Finally, articles that were not in English were excluded from this study due to time and cost constraints. This limits our findings in terms of generalizability, the lack of culturally specific issues, as well as constraints such as a severe lack of funding in developing countries [77]. Future research could strengthen our understanding of these barriers by incorporating translated studies from low-resource settings.

Future research directions

Currently, strong evidence exists within individual specialties regarding lead protection and radiation exposure. However, future research should focus on developing a unified, cross-specialty standard protocol for radiation safety that can be widely applied and evaluate its effect on adherence to safety guidelines. Increased radiation exposure is linked to increased cancer risk; however, only orthopedic surgeons showed higher-than-expected cancer prevalence compared to other specialties [63]. Future multi-specialty cancer prevalence studies could (1) limit participants to those frequenting intraoperative fluoroscopy (i.e. hand hand-specific plastic surgeons rather than all plastic surgeons) and (2) further investigate the education and compliance to radiation safety standards of other subspecialty fields, as surgeon awareness and education are protective factors against radiation exposure [23].

Orthopedic surgeons have a higher infertility, miscarriage, and pregnancy complications compared to the general U.S. population; however, this correlation to intraoperative fluoroscopy remains unstudied [70]. Future infertility impact research should include radiation exposure levels of female surgeons. Bowman et al. and Duggan et al. showed that increasing access to lead shielding led to higher rates of protective equipment use among residents [23,25]. Given evidence that compliance drops when surgeons are required to supply or locate their own PPE [23], future research should identify and highlight programs that actively safeguard their residents. Additionally, because safety is a multidisciplinary responsibility, future studies should emphasize education for all operating room staff, implementing interventions across all shifts, and ensuring knowledge retention despite high turnover.

Finally, attention should be given to the risks of direct radiation exposure when a surgeon’s extremity must be positioned within the fluoroscopy field, as well as the benefits of newer lead protection devices, such as axillary shields, to operating surgeons.

## Conclusions

Fluoroscopy is an effective but potentially hazardous modality that is commonly used across surgical practices. Via systematic literature review, we have developed a multispecialty overview detailing the radiation exposure, resultant risk, and protective methods pertinent to surgeons who utilize fluoroscopy.
